# Nine treatments of 1000 mL therapeutic phlebotomy in a subject with polycythemia: A case report

**DOI:** 10.14814/phy2.16035

**Published:** 2024-06-06

**Authors:** William J. Morton, Anton Hauge, Helge Opdahl, Erling Bekkestad Rein, Signe Søvik, Jonny Hisdal

**Affiliations:** ^1^ Department of Anaesthesia Akershus University Hospital Lørenskog Norway; ^2^ Institute of Clinical Medicine Faculty of Medicine University of Oslo Oslo Norway; ^3^ Department of Vascular Surgery Oslo University Hospital Oslo Norway; ^4^ Department of Acute Medicine, The Norwegian National CBRNE Medical and Advisory Centre Oslo University Hospital Oslo Norway

**Keywords:** hemorrhage, human, phlebotomy, polycythemia

## Abstract

Large‐volume therapeutic phlebotomy is the mainstay of hemochromatosis treatment and offers an opportunity to investigate the hemodynamic changes during acute hypovolemia. An otherwise healthy 64‐year‐old male with hemochromatosis participated. On nine separate visits, 1000 mL therapeutic phlebotomy was performed. On one occasion, pre‐ and post‐phlebotomy orthostatic challenge with 27° reverse Trendelenburg position was administered. Mean arterial pressure, heart rate, and stroke volume were measured continuously during the procedures. The patient's tolerance to the interventions was continuously evaluated. The procedures were well tolerated by the patient. Mean arterial pressure was maintained during hemorrhage and following phlebotomy in both supine and reverse Trendelenburg positions, primarily through an increase in heart rate and systemic vascular resistance. The present study found that 1000 mL therapeutic phlebotomy in a patient with hemochromatosis may be acceptably and safely used to model hemorrhage. The approach demonstrates high clinical applicability and ethically robustness in comparison with volunteer studies.

## 1 INTRODUCTION

Hemochromatosis is characterized by elevated hematocrit and hemoglobin. Increased mortality is largely owed to solid‐organ iron deposition and hyperviscosity syndrome (Murphree et al., 2020). Therapeutic phlebotomy (TP) has the goal of normalizing the hematocrit and ameliorating potential complications (Sloop et al., 2015).

Acute hemorrhage is of clinical and research importance (Shah et al., 2023). The ethical and practical challenges of studying clinical acute hemorrhage have resulted in the development of lower body negative pressure (LBNP) to reversibly simulate central hypovolemia (Goswami et al., 2019). Although LBNP has been found to model the hemodynamics of actual blood loss (Johnson et al., 2014), TP has advantages compared to LBNP. First, blood loss is real rather than a redistribution of blood to the lower extremities. Second, the hemochromatosis patient population requiring phlebotomy better reflects the clinical population, since it consists of individuals aged over 40 rather than the healthy volunteers commonly used in LBNP studies.

Volunteers undergoing LBNP studies do so for presumably do altruistic reasons. In TP, patients´ experimentation is secondary to their clinical treatment, rather than the *principale propositum*. Section 17 of the WMA Declaration of Helsinki (World Medical Association, 2013) states that “All medical research involving human subjects must be preceded by careful assessment of predictable risks and burdens to them.” A study investigating blood loss during TP in a hemochromatosis patient will cause minimal extra burdens, conversely in a volunteer any procedure may be considered burdensome. Thus, hemochromatosis patients can be considered to have limited added burden associated with the research and receive the benefit of treatment, while volunteers have considerable burdens and no benefits.

We hypothesized that study of the hemodynamic effects of acute blood loss in therapeutic phlebotomy may offer an ethical alternative and greater clinical applicability than LBNP studies in healthy volunteers. This historical case series aims to determine if acute hemorrhage, including an orthostatic challenge, may be adequately modeled in a hemochromatosis patient requiring repeated therapeutic phlebotomy.

## 2 METHODS

Following approval by the Regional Ethics Committee (RKMFII ref S‐96203) and informed written consent, an otherwise healthy Caucasian 64‐year‐old man with homozygous hemochromatosis requiring repeated phlebotomy was recruited. Clinical examination and echocardiogram revealed a functionally normal heart.

The tolerability of rapid hemorrhage was assessed by titrating the volume of phlebotomy, from 500 to 1000 mL over three visits. 1000 mL blood loss was well tolerated by the subject, and this volume was subsequently used.

Between 1997 and 2003, the hemodynamic measures associated with clinically indicated 1000 mL phlebotomy were investigated on nine instances. Each intervention was followed with an infusion of 1000 mL of Ringer acetate.

Following local anesthetic, the femoral vein was catheterized. Cardiovascular measurements were continuously recorded. Using a 50 mL syringe and three‐way tap, 1000 mL blood was aspirated over a period of 11–15 min (mean 742 s, range 659–910 s).

At the participant's suggestion and with ethics committee approval, we exacerbated the post TP hemodynamic stress with reverse Trendelenburg position (RTP) (Matzen, 1995). Comparing RTP responses before and after acute blood loss has clinical implications for hypovolemic patients in head up positions, for example, on the operating table. One episode of tilt table testing was conducted. Cardiovascular measurements were recorded during the supine (120 s) and RTP (27° head up, 120 s) positions; then, 1000 mL phlebotomy was performed followed by measurements in the supine and RTP positions.

### 2.1 Measures

Beat to beat cardiac stroke volume (SV) was recorded continuously with ultrasound Doppler, using a 2 MHz probe (SD‐100, GE Vingmed Ultrasound, Horten, Norway) (Eriksen & Walløe, 1990).

Three lead ECG heart rate (HR) and the ultrasound maximum velocity signal were interfaced to a personal computer. The diameter of the aortic ring was determined (CFM‐750, GE Vingmed Ultrasound, Horten, Norway) and used to calculate the aortic valvar root area. SV was calculated by integration of the recorded maximum velocity during each R‐R cycle and the area of the aortic root (Eriksen & Walløe, 1990). Cardiac output (CO) was calculated as HR × SV.

Blood pressure was continuously measured using the volume‐clamp method (Finapres BP monitor; Ohmeda, Madison, WI USA) (Imholz et al., 1998). Mean arterial pressure (MAP) was calculated by numerical integration. Systemic vascular resistance (SVR) was calculated as MAP/CO.

Beat to beat values were calculated for all variables and gated by the ECG R‐wave. The supine position was defined as an angle of <1° from the horizontal plane and RTP as an angle >25°.

JMP® Pro 17.0.0 (JMP Statistical Discovery LLC) was used for statistical analysis. Data are medians and interquartile ranges (IQR) if not otherwise stated. Wilcoxon signed rank test was used for paired comparisons pre–post blood loss. Results were interpreted as statistically significant with a *p* value < 0.05.

The CARE checklist was used in the preparation of this article (Riley et al., 2017).

## 3 RESULTS

The procedures were well tolerated by the patient.

### 3.1 Central hemodynamic responses

**FIGURE 1:**
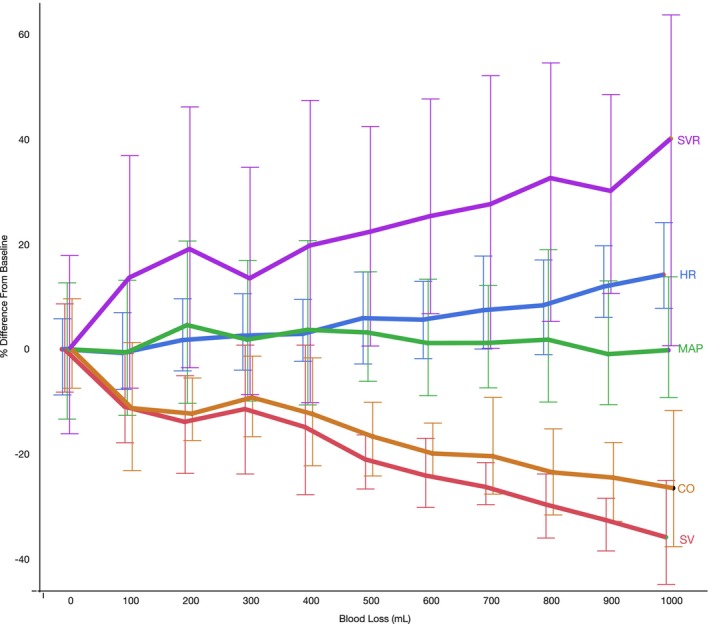
Hemodynamic variables versus blood loss during therapeutic phlebotomy, shown as medians and interquartile ranges. Heart rate (HR) and mean arterial blood pressure (MAP) data represent nine instances; stroke volume (SV), cardiac output (CO), and systemic vascular resistance (SVR) data six instances.

HR and blood pressure data were available from nine episodes of phlebotomy. Concurrent ultrasound measurements were available from six of these episodes. Increasing blood loss was associated with a marked decrease in SV, counteracted by a moderate increase in HR and marked increase in SVR. Consequently, CO declined while MAP and systolic arterial pressure was maintained at pre‐intervention levels during hemorrhage through an increase in HR and SVR (Figure 1). Absolute and percentage changes in cardiovascular variables after 1000 mL blood withdrawal are presented in Table 1.

**TABLE 1:** Hemodynamic changes resulting from a 1000 mL therapeutic phlebotomy.

	*N*	Baseline median (IQR)	Median change (IQR)	Percentage change (IQR)	*p* Value
Heart rate (beats/min)	9	75 (68–77)	8.3 (6.1 to 13.7)	+11.0 (8.5 to 18.1)	0.0039*
Stroke volume (mL/beat)	6	86.6 (76.5–90.5)	−30.3 (−38.3 to −21.2)	−33.1 (−45.2 to −26.1)	0.0313*
Cardiac output (L/min)	6	6.3 (5.7–6.7)	−1.4 (−2.4 to 0.9)	−23.1 (−36.0 to −14.3)	0.0313*
Mean arterial pressure (mmHg)	9	87 (78–93)	0.5 (−4.8 to 3.6)	+0.5 (−5.4 to 4.0)	1.000
Systolic blood pressure (mmHg)	9	122 (108–135)	−5.7 (−9.9 to 5.0)	−4.2 (−7.7 to 4.0)	0.2031
Diastolic blood pressure (mmHg)	9	70 (62–72)	2.7 (0.3 to 5.6)	+3.9 (0.4 to 9.3)	0.0430*
Systemic vascular resistance (mmHg/[L/min])	6	13.3 (11.3–15.9)	3.79 (1.38 to 9.0)	+35.5 (10.5 to 68.0)	0.0313*

*Note*: *p* Values calculated by Wilcoxon signed rank test, two‐sided.

Abbreviation: IQR, interquartile range.

* *p* < 0.05.

### 3.2 Changes from baseline after 1000 mL blood loss

#### 3.2.1 Hemodynamic response to reverse Trendelenburg position

Values of SV, CO, MAP, and SVR during RTP pre‐phlebotomy closely resembled supine values found after 1000 mL blood loss. Compared with RTP pre‐phlebotomy, RTP post‐phlebotomy induced an accentuated HR increase, slightly less further declines in SV and CO, similar increase in SVR, and a smaller increase in MAP (Table 2).

**TABLE 2:** Hemodynamic variables from one episode of Supine and 27° reverse Trendelenburg position (RTP) in a patient with hemochromatosis undergoing therapeutic phlebotomy.

	Pre‐phlebotomy			Post‐phlebotomy		
	Supine median (IQR)	RTP median (IQR)	Mean difference (95% CI)	Supine median (IQR)	RTP median (IQR)	Mean difference (95% CI)
Heart rate (beats/min)	76.8 (76.6–77.1)	79.1 (78.3–79.4)	1.85 (1.7 to 2.0)*	77.3 (76.9–77.8)	84.7 (82.8–85.2)	6.27 (5.9 to 6.6)*
Stroke volume (mL/beat)	78.7 (77.1–80.3)	60.2 (58.0–61.7)	−18.64 (−19.4 to −17.9)*	62.4 (60.0–65.1)	45.3 (44.3–46.5)	−16.96 (−17.7 to −16.2)*
Mean arterial pressure (mmHg)	78.3 (77.7–79.0)	86.7 (85.8–88.0)	8.51 (8.2 to 8.8)*	87.0 (86.3–88.0)	89.5 (88.4–91.2)	2.88 (2.5 to 3.3)*
Systemic vascular resistance (mmHg/[L/min])	13.0 (12.7–13.3)	18.3 (17.7–19.2)	5.43 (5.3 to 5.6)*	18.0 (17.4–18.8)	23.3 (22.7–24.6)	5.26 (5.3 to 5.8)*
Cardiac output (L/min)	6.1 (5.9–6.2)	4.8 (4.5–4.9)	−1.32 (−1.4 to −1.3)*	4.8 (4.6–5.0)	3.8 (3.7–3.9)	−1.03 (−1.1 to −1.0)*

*Note*: Post‐phlebotomy = post 1000 mL blood loss. Mean difference: between supine and RTP positions.

Abbreviation: IQR, interquartile range.

* *p* < 0.001 for comparison Supine versus RTP.

## 4 DISCUSSION

In this study, 1000 mL acute blood loss was well tolerated, and the hemodynamic compensation could be characterized in detail. MAP remained within the baseline range in the supine position and increased during RTP, while SV decreased. The SV related decrease in CO was compensated for by an increase in HR and SVR, as previously described (Kirkman & Watts, 2014). Hemodynamic responses to RTP differed according to volume state (pre‐ vs. post‐phlebotomy). The mechanisms by which our subject compensated to such a high degree may include (1) conditioning by exposure to repeated phlebotomy, (2) a high hematocrit, which is the major determinant of blood viscosity and consequently elevated SVR (Pearson et al., 1980). Visual inspection of data did not reveal time trends. In this study, hematocrit should be considered a relatively constant parameter since there was little time for autologous expansion of the plasma volume to occur following phlebotomy.

Having the blood loss occur over 11–15 min increases this study's applicability to clinical scenarios in which there is rapid bleeding. The subject's age (64 years) further increases the clinical applicability. There is an increased prevalence and risk of trauma in the elderly (Adams & Holcomb, 2015), who also more frequently use anticoagulant therapy. Elderly patients may respond differently from younger patients (Kuchel et al., 1992) and warrant targeted research.

## 5 CONCLUSION

The present study found that 1000 mL therapeutic phlebotomy in a patient with hemochromatosis may be acceptably and safely used to model hemorrhage. The approach demonstrates high clinical applicability and ethically robustness in comparison with volunteer studies.

## FUNDING INFORMATION

The study was funded by Akershus University Hospital, the Institute of Basic Medical Sciences, and the Institute of Clinical Medicine, University of Oslo, Norway.

## CONFLICT OF INTEREST STATEMENT

The subject of this study is also one of the investigators and co‐authors.

## DISCLOSURES

No generative artificial intelligence tools were used in the preparation of this manuscript.

## Data Availability

The data that support the findings of this study are available from the corresponding author on request. The data are not publicly available due to privacy or ethical restrictions.

## References

[phy216035-bib-0001] Adams, S. D. , & Holcomb, J. B. (2015). Geriatric trauma. Current Opinion in Critical Care, 21, 520–526. 26539925 10.1097/MCC.0000000000000246

[phy216035-bib-0002] Eriksen, M. , & Walløe, L. (1990). Improved method for cardiac output determination in man using ultrasound doppler technique. Medical & Biological Engineering & Computing, 28, 555–560. 2287179 10.1007/BF02442607

[phy216035-bib-0003] Goswami, N. , Blaber, A. P. , Hinghofer‐Szalkay, H. , & Convertino, V. A. (2019). Lower body negative pressure: Physiological effects, applications, and implementation. Physiological Reviews, 99, 807–851. 30540225 10.1152/physrev.00006.2018

[phy216035-bib-0004] Imholz, B. P. , Wieling, W. , Van Montfrans, G. A. , & Wesseling, K. H. (1998). Fifteen years experience with finger arterial pressure monitoring: Assessment of the technology. Cardiovascular Research, 38, 605–616. 9747429 10.1016/s0008-6363(98)00067-4

[phy216035-bib-0005] Johnson, B. D. , Van Helmond, N. , Curry, T. B. , VAN Buskirk, C. M. , Convertino, V. A. , & Joyner, M. J. (2014). Reductions in central venous pressure by lower body negative pressure or blood loss elicit similar hemodynamic responses. Journal of Applied Physiology, 1985(117), 131–141. 10.1152/japplphysiol.00070.2014 PMC4459917 24876357

[phy216035-bib-0006] Kirkman, E. , & Watts, S. (2014). Haemodynamic changes in trauma. BJA: British Journal of Anaesthesia, 113, 266–275. 25038158 10.1093/bja/aeu232

[phy216035-bib-0007] Kuchel, G. A. , Avorn, J. , Reed, M. J. , & Fields, D. (1992). Cardiovascular responses to phlebotomy and sitting in middle‐aged and elderly subjects. Archives of Internal Medicine, 152, 366–370. 1739368

[phy216035-bib-0008] Matzen, S. H. (1995). Neuroendocrine mechanisms during reversible hypovolaemic shock in humans with emphasis on the histaminergic and serotonergic system. Acta Physiologica Scandinavica. Supplementum, 628, 1–31. 8801774

[phy216035-bib-0009] Murphree, C. R. , Nguyen, N. N. , Raghunathan, V. , Olson, S. R. , Deloughery, T. , & Shatzel, J. J. (2020). Diagnosis and management of hereditary haemochromatosis. Vox Sanguinis, 115, 255–262. 32080859 10.1111/vox.12896

[phy216035-bib-0010] Pearson, T. C. , Ring, C. P. , & Wetherley‐Mein, G. (1980). Plasma and whole blood viscosity in treated primary polycythaemia. Clinical and Laboratory Haematology, 2, 73–82. 7379470 10.1111/j.1365-2257.1980.tb00810.x

[phy216035-bib-0011] Riley, D. S. , Barber, M. S. , Kienle, G. S. , Aronson, J. K. , Von Schoen‐Angerer, T. , Tugwell, P. , Kiene, H. , Helfand, M. , Altman, D. G. , Sox, H. , Werthmann, P. G. , Moher, D. , Rison, R. A. , Shamseer, L. , Koch, C. A. , Sun, G. H. , Hanaway, P. , Sudak, N. L. , KASZKIN‐Bettag, M. , … Gagnier, J. J. (2017). CARE guidelines for case reports: Explanation and elaboration document. Journal of Clinical Epidemiology, 89, 218–235. 28529185 10.1016/j.jclinepi.2017.04.026

[phy216035-bib-0012] Shah, A. , Kerner, V. , Stanworth, S. J. , & Agarwal, S. (2023). Major haemorrhage: Past, present and future. Anaesthesia, 78, 93–104. 36089857 10.1111/anae.15866 PMC10087440

[phy216035-bib-0013] Sloop, G. , Holsworth, R. E. , Weidman, J. J. , & ST Cyr, J. A. (2015). The role of chronic hyperviscosity in vascular disease. Therapeutic Advances in Cardiovascular Disease, 9, 19–25. 25260890 10.1177/1753944714553226

[phy216035-bib-0014] World Medical Association . (2013). WMA declaration of Helsinki—Ethical principles for medical research involving human subjects. *64th WMA General Assembly*. Retrieved November 4, 2020, from https://www.wma.net/policies‐post/wma‐declaration‐of‐helsinki‐ethical‐principles‐for‐medical‐research‐involving‐human‐subjects/

